# Expression of porcine protegrin-1 in *Pichia pastoris* and its anticancer activity *in vitro*

**DOI:** 10.3892/etm.2015.2202

**Published:** 2015-01-22

**Authors:** MINGFU NIU, SHUMAO CHAI, XIAOYAN YOU, WENHUI WANG, CUILI QIN, QIANG GONG, TINGTING ZHANG, PENG WAN

**Affiliations:** Food and Bioengineering College, Henan University of Science and Technology, Luoyang, Henan 471003, P.R. China

**Keywords:** antibacterial peptide protegrin-1/His, expression and purification, anticancer activity

## Abstract

Protegrin-1 (PG-1), a β-hairpin antimicrobial peptide (AMP), is amongst the shortest AMPs in sequence length while remaining active against a variety of microorganisms. The aim of this study was produce recombinant PG-1 and investigate its anticancer activity. A DNA sequence encoding the mature PG-1, fused with a 6His-tag, was cloned into the pPICZα-A vector and transformed into *Pichia pastoris*. Expression was induced following culture for ~96 h with 1% methanol at 28°C, and ~15.6 mg PG-1 was expressed in 100 ml culture medium. Following purification using a Ni-chelating Sepharose column, ~20 mg pure active PG-1 was obtained from 500 ml culture broth supernatant. The expressed PG-1/6His exhibited strong dose- and time-dependent anticancer activity against HepG2 cells *in vitro*.

## Introduction

Antimicrobial peptides (AMPs) are significant components of the innate immune systems of numerous animal species, where they act as an effective, largely non-discriminatory first line of defense against invading pathogens (1). A variety of AMPs have been identified from a variety of sources, ranging from plants and insects to lower vertebrates and mammals, as described in previous studies (2,3).

Protegrin-1 (PG-1) is a β-hairpin AMP of 18 amino acids (RGGRLCYCRRRFCVCVGR) that was originally isolated from porcine leukocytes (4). PG-1 is rich in cationic residues, such as arginine (Arg). The amphipathic characteristic of this peptide enables it to interact with the membranes of pathogens and kill the pathogen by releasing its cellular contents (5). Studies have revealed a number of the key steps in the action of protegrin, including: Protegrin monomers dimerize in various types of lipid environment; protegrin peptides interact strongly with lipid bilayer membranes, particularly those that contain anionic lipids; and protegrins form pores in lipid bilayers, which results in uncontrolled ion transport and may be a key factor in bacterial death (1). PG-1 is considered as a potential pharmaceutical agent as it has shown a broad range of antimicrobial activities against gram-positive and gram-negative bacteria, including *Escherichia coli* (*E. coli*), *Pseudomonas aeruginosa* and *Neisseria gonorrhoeae* (5–7).

For pharmaceutical applications, a large quantity of AMP is required. Chemical synthesis of AMPs is not economically practical, particularly for the production of long peptides. Thus, a biological expression system would be an improved alternative method of production (2). Numerous biological expression systems have been introduced for the economical production of AMPs (8). For the production of recombinant AMPs, *E. coli*-based systems are well established (8,9) and *Pichia pastoris* (*P. pastoris*) expression systems are frequently used (10–14). However, each system has limitations, including poor recovery yields, proteolysis of products, low expression levels, toxicity of the product to host cells, and occasionally an absence of the post-translational modifications required for the biological activity of the AMPs (2,8,15).

The aim of the present study was to express the antimicrobial peptide PG-1 in *P. pastoris* X-33, isolate the recombinant product and investigate its anticancer activity.

## Materials and methods

### Strains, vector, enzymes and other reagents

*E. coli* DH5α (Shanghai Sangon Biological Engineering Co. Ltd., Shanghai, China) was used for plasmid amplification and *P. pastoris* X-33 was used for the expression of the fusion protein. The pPICZα-A expression vector and *P. pastoris* X-33 were purchased from Invitrogen Life Technologies (Carlsbad, CA, USA). The restriction endonuclease, T4 DNA ligase and Taq DNA polymerase were purchased from Takara Biotechnology Co., Ltd. (Dalian, China). Ni-chelating Sepharose columns were purchased from Shanghai Sangon Biological Engineering Technology and Services Co., Ltd. (Shanghai, China).

### Cell culture

The HepG2 cell line was obtained from the Medical College of Henan University of Science and Technology (Luoyang, China). The cells were grown in Dulbecco’s modified Eagle’s medium (DMEM; Shanghai Sangon Biological Engineering Co. Ltd.) supplemented with 10% heat-inactivated fetal bovine serum, 100 U/ml penicillin and 100 μg/ml streptomycin in a humidified 5% CO_2_ atmosphere at 37°C.

### Design and synthesis of the PG-1 nucleotide sequence

**Figure 1:**
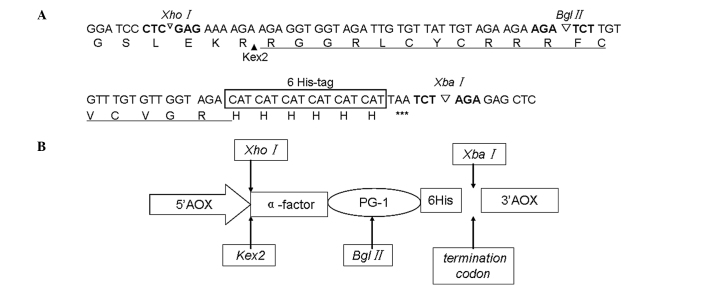
(A) Synthetic cDNA sequence of PG-1 and the corresponding amino acid sequence. The amino acid sequence of the mature PG-1 is underlined. The amino acid sequence of the host enzyme Kek2 cleavage site necessary for proteolytic processing of the *Saccharomyces cerevisiae* α-factor sequence is indicated by a black arrow. The *Xho*I, *Bgl*II and *Xba*I restriction sites are shown in bold. The added 6His-tag sequence is shown in-frame. The termination codon is marked with three asterisks. PG-1, protegrin-1. (B) Site of the PG-1 gene in frame with the α-factor secretion signal of the pPICZα shuttle vector A.

Based on the primary amino acid sequences of the mature peptide and according to codon preference of *P. pastoris* X-33, the gene sequence encoding PG-1 (Fig. 1A) was synthesized and used to construct the plasmid pUC57-PG-1 (Shanghai Sangon Biological Engineering Co. Ltd.). An *Xho*I restriction enzyme site was introduced to allow in-frame cloning into the α-factor secretion signal of the pPICZα-PG-1 shuttle vector. A sequence encoding the Kex2 cleavage site (LEKR) and a 6His-tag were added upstream and downstream of the PG-1 codon sequence, respectively. A termination codon of PG-1 was introduced at the C-terminus, along with a *Xba*I restriction enzyme site (Fig. 1B).

### Construction of the expression vectors

With the plasmid pUC57-PG-1 as the template, the gene encoding PG-1 was amplified by polymerase chain reaction (PCR) with the common primers pUC57+ (5′-ATCAGGCGCCATTCGCCATTC-3′) and pUC57- (5′-CAGGTTCCCGACTGGAAAG-3′). The PCR mixture had a total volume of 50 μl and comprised 5 μl 10X PCR buffer, 4 μl dNTP mixture, 1 μl 20 μl/ml primer pUC57+, 1 μl 20 μl/ml primer pUC57-, 2 μl template, 1 μl Taq DNA polymerase and 36 μl ddH_2_O. The PCR procedure involved 4 min predenaturation at 95°C; 30 sec denaturation at 94°C, 30 sec annealing at 53°C and 30 sec extension at 72°C for 30 cycles; followed by 3 min extension at 72°C. The amplified products were verified by agarose gel electrophoresis. The PCR products were and the pPICZα A plasmid were double digested with XhoI and *Xba*I respectively, and ligated with T4 DNA ligase to generate the fusion vector pPICZα-A. The plasmid was then digested with the same restriction enzymes, to generate the fusion vector pPICZα-A-PG-1. The ligation mixture was transformed into *E. coli* DH5α, and recombinant *E. coli* cells were selected on Zeocin-containing lysogeny broth plates (Invitrogen Life Technologies). The recombinant plasmid pPICZα-A-PG-1 was confirmed by restriction endonuclease digestion and DNA sequencing analysis.

### Transformation of P. pastoris and selection of transformants

pPICZα-A-PG-1 was linearized with *Sac*I and transformed into competent cells of *P. pastoris* X-33 (Mut^+^) by electroporation (Gene Pulser Xcell™ Electroporation System; Bio-Rad, Hercules, CA, USA), according to the manufacturer’s instructions (*Pichia* Expression kit; Invitrogen Life Technologies). The empty pPICZα-A vector was similarly linearized and transformed into *P. pastoris* X-33 as a negative control. All Zeocin-resistant colonies growing in Yeast Extract Peptone Dextrose medium (1% yeast extract, 2% peptone, 2% dextrose, 1 M sorbitol, 2% agar and 100 mg/ml Zeocin) plates were plated in duplicate onto either minimal methanol with histidine [MMH; 1.34% yeast nitrogen base (YNB), 0.05% biotin, 0.5% methanol and 1.5% agar] or minimal dextrose with histidine (MDH; 1.34% YNB, 0.05% biotin, 1% dextrose and 1.5% agar) plates to characterize the methanol-utilizing phenotype. Mut^+^ strains were obtained from the MDH plates and the inserts were evaluated by PCR amplification of the yeast genomic DNA template, using the common aldehyde oxidase 1 primers (5′-GACTGGTTCCAATTGACAAGC and 5′-GCAAATGGCATTCTGACATCC). These strains were subsequently used for the suspension culture.

### Heterologous expression of recombinant PG-1 in P. pastoris X-33

**Figure 2:**
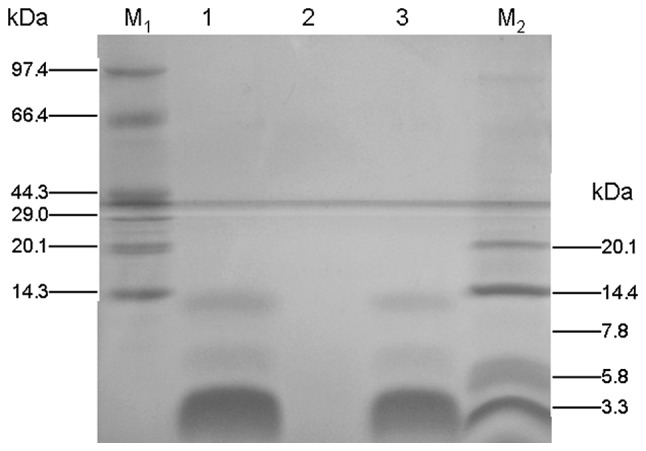
Tricine-SDS-PAGE analysis of the recombinant PG-1 in fermentation supernatants from *P. pastoris* in shaker flask cultures. M1, high range protein marker; M2, low molecular weight marker; lanes 1 and 3, samples from PG-1-expressing *P. pastoris* after 4 days of methanol induction; and lane 2, medium from culture containing PG-1-free *P. pastoris* 4 days after methanol treatment. Tricine-SDS-PAGE, Tricine-sodium dodecyl sulfate-polyacrylamide gel electrophoresis; PG-1, protegrin-1; *P. pastoris*, *Pichia pastoris*.

The positive *P. pastoris* transformants were selected and inoculated in 10 ml buffered glycerol-complex medium (1% yeast extract, 2% peptone, 100 mM potassium phosphate buffer pH 6.0, 1.34% YNB, 4×10^−5^% biotin and 1% glycerol) for 24 h at 28°C. When the cell density reached ~0.6 at optical density (OD)600 (725 UV Visible Spectrophotometer; Shanghai Precision & Scientific Instrument Co., Ltd., Shanghai, China), the cells were harvested by centrifugation at 3,000 × g for 2 min and resuspended to an OD600 of 1.0 in buffered methanol-complex medium (1% yeast extract, 2% peptone, 100 mM potassium phosphate buffer pH 6.0, 1.34% YNB, 4×10^−5^% biotin and 1.0% methanol). The cells were then cultured for 4–5 days at 28°C in a flask while adding methanol to a final concentration of 1% every 24 h. The cell culture medium was harvested by centrifugation at 12,000 × g for 20 min. The presence of the fusion protein in the supernatant was analyzed by Tricine-sodium dodecyl sulfate-polyacrylamide gel electrophoresis (Tricine-SDS-PAGE) with Coomassie Brilliant Blue staining (16). As shown in Fig. 2, a major protein recombinant PG-1 band of ~2.9 kDa appeared following induction for 4 days. The protein concentration was determined using Bio-Rad Protein Assay Dye Reagent with bovine serum albumin (BSA) as standard (17).

### Purification of peptide PG-1 and concentration determination

The expression supernatant was applied to a Ni-chelating Sepharose column (1.6×10 cm) pre-equilibrated with phosphate-buffered saline to purify the PG-1 fusion protein. Subsequently, the column was washed with 20 bed volumes of the same buffer to remove contaminating proteins. The protein was eluted with a linear gradient from 0.1 to 1.0 M imidazole in 1X Ni-NTA elution buffer (50 mM NaH_2_PO_4_ pH 8.0 and 300 mM NaCl). The peak amount of PG-1 eluted was determined by analysis of the fractions with 15% Tricine-SDS-PAGE, and the fractions containing PG-1 were collected. The collected fractions were dialyzed in Milli-Q water (Millipore, Billerica, MA, USA). The protein concentration of the purified PG-1 was determined using Bio-Rad Protein Assay Dye Reagent with BSA as standard. Subsequently, the purified peptide was lyophilized and stored at −20°C until use.

### In vitro assay for cytotoxic activity

The cytotoxicity of the purified PG-1 fusion protein was determined by a tetrazolium (MTT) assay (18). The HepG2 cells (3×10^3^ cells/well) were plated in 100 μl DMEM per well in 96-well plates (Costar Corning, Corning, NY, USA). Following overnight incubation, purified PG-1 was added in various concentrations (10, 20, 40, 60 and 160 μg/ml) to the wells, with six wells for each concentration. After treatment with purified PG-1 for 1, 2, 3, 4 and 5 days, 20 μl 5 mg/ml MTT (pH 4.7) was added to each well and the cells were cultivated for a further 4 h. The supernatant fluid was then removed, 100 μl DMSO was added to each well and the samples were agitated for 15 min. The absorbance at 490 nm was measured with a microplate reader (Bio-Rad) using wells without cells as blanks. Three independent experiments were performed for each set of conditions.

## Results

### Construction of the recombinant plasmid pPICZα-A-PG-1

Using degenerate primers, the PG-1 gene was amplified by PCR and verified by agarose gel electrophoresis. The PCR product was ligated into the pPICZα-A expression vector together with an α-mating factor secretion signal sequence at the N-terminus and a 6His-tag at the C-terminus of the PG-1 peptide. The recombinant plasmid pPICZα-A-PG-1 was successfully constructed and verified by restriction enzyme analysis and DNA sequencing.

### Recombinant pPICZα-A-PG-1 expression and purification

**Figure 3:**
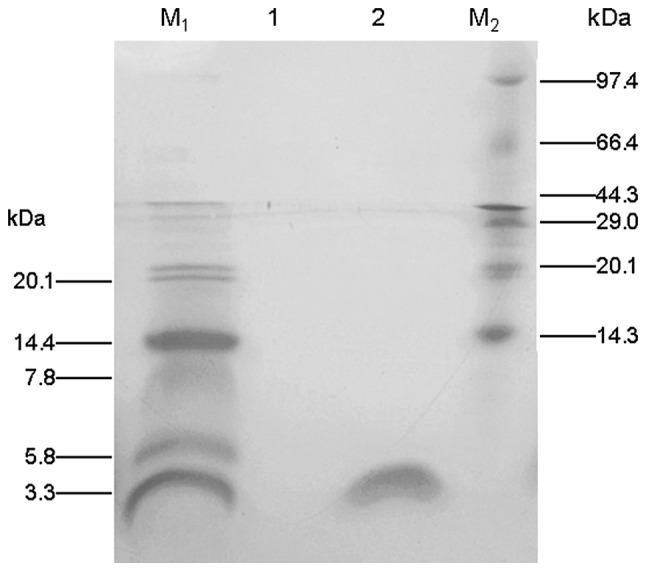
Tricine-SDS-PAGE analysis of the PG-1 purified by Ni^2+^ affinity chromatography. M1 and M2, protein molecular weight markers; lane 1, PBS wash-out column, and lane 2, purified PG-1. Tricine-SDS-PAGE, Tricine-sodium dodecyl sulfate-polyacrylamide gel electrophoresis; PG-1, protegrin-1.

The recombinant plasmid pPICZα-A-PG-1 was linearized with *Sac*I and transformed into competent cells of *P. pastoris* X-33. The pPICZα-A-PG-1 transformants of *P. pastoris* were grown in flasks at 28°C and, after culture for 120 h, the cell culture medium was harvested by centrifugation. The supernatant from the flask culture was analyzed by Tricine-SDS-PAGE. As shown in Fig. 2, a major band at ~2.9 kDa was observed after a 96-h induction. The protein concentration in the supernatent was 15.6mg/100ml, as measured using Bio-Rad Protein Assay Dye Reagent Using an Ni-NTA column, the fusion protein was eluted at 250 mM imidazole. The eluted fractions were collected and dialyzed, and the results of the Tricine-SDS-PAGE (Fig. 3) indicated that the recombinant PG-1 fusion protein (2.9 kDa) had been obtained with high-purity. Approximately 20 mg PG-1/6His was purified from 500 ml culture medium.

### Cytotoxic activity of PG-1 against HepG2 cells

**Figure 4:**
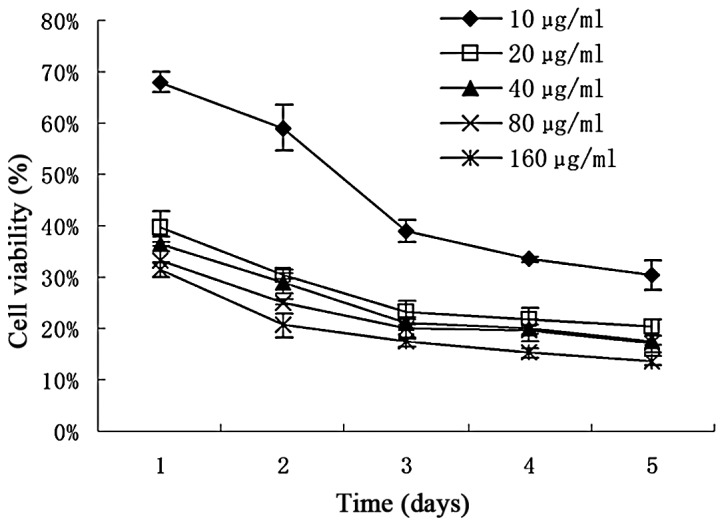
Inhibition of the growth of HepG2 cells by 6His-tagged PG-1. The cells were seeded onto 96-well plates at a density of 3×10^3^ cells/well and treated with PG-1 at different concentrations. The percentage of cell viability was determined by an MTT assay after 1, 2, 3, 4 and 5 days of treatment. Dose- and time-dependent growth inhibition was observed at the concentrations between 20 and 160 μg/ml. The results are the mean values ± standard deviation of three independent experiments, each in six wells. PG-1, protegrin-1.

The results of the *in vitro* assay of the cytotoxic activity of the PG-1 fusion protein against HepG2 cells are shown in Fig. 4. The percentage of growth inhibition of the HepG2 cells by PG-1/6His at various concentrations was determined by the number of viable treated cells in comparison with the number of viable cells of the untreated controls. The results showed that PG-1/6His had a dose- and time-dependent inhibitory effect on cell growth.

## Discussion

PG-1 (H_3_N^+^-RGGRLCYCRRRFCVCVGR-CONH_2_), with a high content of positively charged Arg and cysteine (Cys) residues, has a β-hairpin structure that is stabilized by disulfide bonds linking Cys-6 and Cys-15, and Cys-8 and Cys-13 (19,20). Its rigid structure separates the hydrophobic and hydrophilic residues of the peptide, resulting in PG-1 having an amphipathic nature that is common to numerous other types of AMP (1,21). PG-1 is highly cationic (charge of 7+) at physiological pH, which is essential for its ability to bind strongly to bacterial cell membranes. It is the amphipathic characteristic of PG-1 that enables it to interact with the membranes of pathogens (4). Protegrin monomers dimerize in various types of lipid environment (1,4,19,21,22). However, the characteristics of the protegrin peptide biologically expressed in the present study, and whether fusion to 6His affects its structure and function are unknown. These factors require investigation in further studies.

The methylotrophic yeast *P. pastoris* has been utilized widely as a heterologous gene expression system. Although PG-1 has been successfully expressed in *E. coli* (8), the expression levels that have been achieved are lower than those obtained for *P. pastoris*-derived PG-1 in the present study. Furthermore, certain recombinant AMPs produced in *P. pastoris* have stronger activity than those produced in *E. coli*, including crab AMP scygonadin (12), plant defensin corn 1 (23), human secretory leukocyte protease inhibitor (24) and non-specific lipid-transfer proteins (25).

In order to facilitate the purification of PG-1 in the present study, a 6His-tag was introduced at the C-terminus of PG-1. A previous study has observed that Mdcec/6His has the same levels of activity against bacteria as those of Mdcec, and has slightly increased activity levels against fungi (11). *P. pastoris*-derived scygonadin/6His has been effectively expressed with higher activity levels against bacteria than those of scygonadin (12). In the present study, the expressed PG-1/6His demonstrated strong dose- and time-dependent anticancer activity against HepG2 cells.
