# [Corrigendum] Expression of porcine protegrin‑1 in *Pichia pastoris* and its anticancer activity *in vitro*

**DOI:** 10.3892/etm.2022.11713

**Published:** 2022-11-18

**Authors:** Mingfu Niu, Shumao Chai, Xiaoyan You, Wenhui Wang, Cuili Qin, Qiang Gong, Tingting Zhang, Peng Wan

Exp Ther Med 9:1075–1079, 2015; DOI: 10.3892/etm.2015.2202

Following the publication of the above article, the authors have realized that they inadvertently uploaded an incorrect figure for Fig. 2 in the article as it appeared on p. 1077, and have requested that this error be rectified through publishing a corrigendum. The Editor has agreed to their request, and the corrected version of Fig. 2, together with its revised figure legend, is shown opposite. Note that the error made in uploading the incorrect version of this figure did not affect the overall conclusions reported in the paper. All the authors agree with the publication of this corrigendum, and are grateful to the Editor of *Experimental and Therapeutic Medicine* for allowing them the opportunity to publish this. They also apologize to the readership for any inconvenience caused.

## Figures and Tables

**Figure 2:**
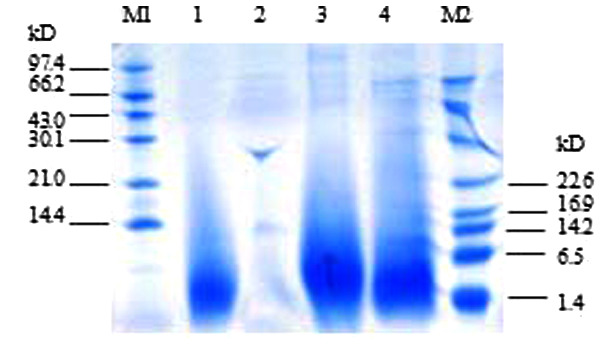
Tricine-SDS-PAGE analysis of the recombinant PG-1 in fermentation supernatants from *P. pastoris* in shaker flask cultures. M1, high range protein marker; M2, low molecular weight marker; lanes 1, 3 and 4, samples from PG-1-expressing *P. pastoris* after 4 days of methanol induction; and lane 2, medium from culture containing PG-1-free *P. pastoris* 4 days after methanol treatment. Tricine-SDS-PAGE, Tricine sodium dodecyl sulfate-polyacrylamide gel electrophoresis; PG-1, protegrin-1; *P. pastoris*, *Pichia pastoris*.

